# Lessons learned: strategies for implementing and the ongoing use of LI-RADS in your practice

**DOI:** 10.1007/s00261-024-04643-8

**Published:** 2024-10-23

**Authors:** Mohammed Ismail, Tasneem Lalani, Ania Kielar, Cheng Hong, Joseph Yacoub, Christopher Lim, Venkateswar Surabhi, Krishna Shanbhogue, Sadhna Nandwana, Xiaoyang Liu, Cynthia Santillan, Mustafa R. Bashir, James Lee

**Affiliations:** 1 The Ohio State University, Columbus, USA; 2 The Ohio State University Wexner Medical Center, Columbus, USA; 3 University of Massachusetts Chan Medical School, Worcester, USA; 4 University of Toronto, Toronto, Canada; 5 University of California San Francisco Medical Center, San Francisco, USA; 6 MedStar Georgetown University Hospital, Washington D.C., USA; 7 Sunnybrook Health Science Centre, Toronto, Canada; 8 The University of Texas MD Anderson Cancer Center, Houston, USA; 9 New York University Langone Medical Center, New York, USA; 10 Emory University, Atlanta, USA; 11 University of California, San Diego, San Diego, USA; 12 Duke Medical Center, Durham, USA; 13 University of Kentucky, Lexington, USA

**Keywords:** HCC, LIRADS, Liver, ACR

## Abstract

The establishment of the Liver Imaging Reporting and Data System (LI-RADS) in 2011 provided a comprehensive approach to standardized imaging, interpretation, and reporting of liver observations in patients diagnosed with or at risk for hepatocellular carcinoma (HCC). Each set of algorithms provides criteria pertinent to the various components of HCC management including surveillance, diagnosis, staging, and treatment response supported by a detailed lexicon of terms applicable to a wide range of liver imaging scenarios. Before its widespread adoption, the variability in the terminology of diagnostic criteria and definitions of imaging features led to significant challenges in patient management and made it difficult to replicate findings or apply them consistently. The integration of LI-RADS into the clinical setting has enhanced the efficiency and clarity of communication between radiologists, referring providers, and patients by employing a uniform language that averts miscommunications. LI-RADS has been strengthened with its integration into the American Association for Study of Liver Diseases practice guidelines. We will provide the background on the initial development of LI-RADS and reasons for development to serve as a starting point for conveying the system’s benefits and evolution over the years. We will also suggest strategies for the implementation and maintenance of a LI-RADS program will be discussed.

## Introduction

The Liver Imaging Reporting and Data System (LI-RADS), established in 2011, is a comprehensive approach to standardized imaging, interpretation, and reporting of liver observations in patients diagnosed with or at risk for hepatocellular carcinoma (HCC). Each set of algorithms provides standardized criteria pertinent to the various components of HCC management including surveillance, diagnosis, staging, and treatment response supported by a detailed lexicon of terms applicable to a wide range of liver imaging scenarios [1, 2]. In clinical practice, the use of non-standardized terminology may lead to misunderstandings among healthcare providers and cause confusion for patients interpreting their medical reports [3]. LI-RADS encourages uniformity of interpretation of complex imaging data allowing for appropriate diagnosis and management using a defined lexicon [4].

The integration of LI-RADS into the clinical setting has enhanced the efficiency and clarity of communication between radiologists, referring providers, and patients by employing a uniform language that averts miscommunications. Studies suggest that the LI-RADS structured reporting template not only enhances the accuracy of the reports but also considerably increases the consistency with which major HCC features are described in radiology reports [2, 4, 5].

By design, LI-RADS extends into scientific research and clinical trials, where its adoption has led to more standardized and consistent definitions across studies, investigating HCC in particular [6]. Prior to its widespread adoption, the variability in diagnostic criteria and definitions of imaging features such as washout and arterial phase hyperenhancement led to challenges in understanding and interpreting study outcomes [3]. Furthermore, variability in scientific terminology complicates the aggregation and analysis of data from existing studies, limiting the process of conducting systematic reviews and meta-analyses, as well as making it difficult to replicate findings or apply them clinically. In addition, LI-RADS has been integrated into the practice guidance of the American Association for Study of Liver Diseases, cementing its role as a standard in HCC management and care [1–3, 7, 8]. Recently, LI-RADS leadership has partnered with the Organ Procurement and Transplantation Network (OPTN) to agree on a concordant definition for LR-5 observations [8].

Nevertheless, implementing LI-RADS in clinical practice can be challenging due to the time investment needed to learn and establish multiple algorithms as well as the resistance to structured reporting. Providing background on the initial development of LI-RADS and reasons for development serves as an excellent starting point for conveying the system’s benefits. Key steps to facilitate the implementation and maintenance of a LI-RADS program will be discussed.

## Introduction to LI-RADS

### Historical context and development

Initial prototypes of LI-RADS were crafted in 2006, aiming to create uniformity in imaging for hepatocellular carcinoma (HCC) across different institutions. In 2008, the American College of Radiology initiated a steering committee to develop these early models into a reporting system [1, 2]. The official release of LI-RADS in 2011 introduced a detailed system for evaluating liver observations on CT and MRI in high-risk adults, drawing inspiration from the Breast Imaging Reporting and Data System (BI-RADS). Akin to BI-RADS, LI-RADS was intended to establish a method for consistent imaging interpretations and well-defined terminology that clearly and accurately categorized the likelihood of malignancy, namely HCC for observations in the liver [1, 7].

The differences in managing liver versus breast malignancies required distinct approaches between LI-RADS and BI-RADS. LI-RADS assigns categories based on individual observations rather than an overall patient assessment as in BI-RADS. While BI-RADS primarily aims to pinpoint lesions that require biopsy, LI-RADS strives for a high degree of specificity in diagnosing HCC, with the LR-5 category signifying definite HCC, obviating the need for biopsy prior to treatment [7, 9].

Since its introduction, LI-RADS has been regularly updated based on user feedback and new data, expanding to include six distinct algorithms: LI-RADS US Surveillance, LI-RADS CEUS for diagnosis, LI-RADS CT/MRI for diagnosis, and three LI-RADS Treatment Response Assessment algorithms [2, 7]. Each algorithm categorizes observations to reflect the probability of disease presence. Complementing these algorithms are supporting documents, a manual, and a lexicon that further elucidates the application of the algorithms and standardized terminology [1, 2, 7].

By 2018, LI-RADS recommendations had been integrated into the American Association for the Study of Liver Diseases’ practice guidelines, and by 2022, LI-RADS achieved concordance with the Organ Procurement and Transplantation Network class 5 criteria. LI-RADS now plays an indispensable role in the care of patients with HCC, as recognized by its inclusion in the AASLD’s Practice Metrics Committee’s quality measures in 2022, reflecting its wide acceptance and application in both academic and community clinical settings worldwide [1, 2, 7].

### Benefits of LI-RADS to motivate implementation of this system in a radiology department

Prior to the introduction of LI-RADS, there was notable variability in how imaging features, such as arterial phase hyperenhancement, were defined, with definitions often being specific to individual studies [6, 7]. Inconsistency in terminology and diagnostic criteria, complicated comparisons across different studies focused on HCC imaging. However, a comparative analysis of the periods before (2011–2013) and after (2017–2019) LI-RADS implementation shows a decline in the variation [7]. Specifically, Anh et al. showed the use of non-LI-RADS study-specific definitions for major features fell from 80 to 25% [6]. This shift has significantly enhanced consistency and the ability to aggregate data across studies [7].

**Fig. 1:**
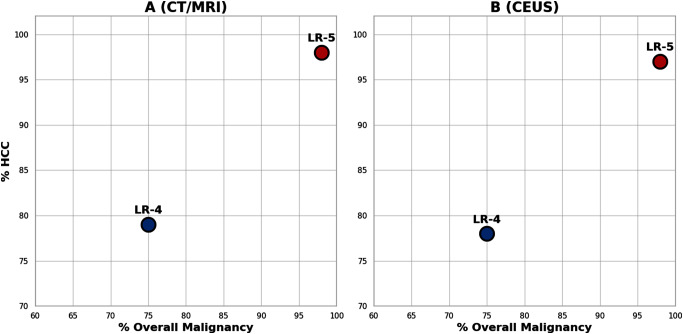
The probabilities of diagnosing HCC and malignancy in the LR-4 (probable HCC) and LR-5 categories using both CT/MRI (graph on the left) and CEUS (graph on the right) are almost identical. Figure adapted from the graphs provided by Chernyak, V et al. [7, 11–13]

The utility of LI-RADS in the clinical setting has been extensively validated. Numerous publications have explored various aspects such as the diagnostic accuracy of different categories and imaging features, consistency among readers, and comparisons between imaging modalities using LI-RADS algorithms. The CT/MRI Diagnostic LI-RADS, being the first and most thoroughly studied algorithm, has seen recent meta-analyses confirm the expected probabilities of diagnostic categories [7]. These analyses demonstrate that the likelihood of HCC increases from LR-1 (definitely benign) to LR-5 (definite HCC), with nearly all findings classified as LR-M (probably or definitely malignant, but not specific to HCC) being malignant [10]. Additionally, the probabilities of diagnosing HCC and malignancy in the LR-4 (probable HCC) and LR-5 categories using both CEUS and CT/MRI are almost identical [7, 11–13] (See Fig. 1).

Although the work to standardize protocols can be initially time consuming, the benefits of reduction in variability of protocols, overall decrease in number of redundant protocols, and the standardization leading to more consistent image acquisition by technologists make it time well spent [14]. The main imaging features of hepatocellular carcinoma include early arterial phase hyperenhancement, compared to background liver parenchyma, as well as washout on portal venous phase or delayed phases, and presence of a capsule and interval growth. The first 3 diagnostic features of HCC are highly dependent on the quality of image acquisition, with a late arterial phase being particular important in CT and MRI imaging. This late arterial phase differs from standard early arterial phase imaging often used for vascular assessment. Thus, adhering to the standard imaging techniques recommended by LI-RADS is a key step in evaluating patients at risk for HCC [15, 16].

## Implementing LI-RADS in clinical practice

### Key strategies to start a LI-RADS program

#### Identify local champions in the department

One key aspect of adopting LI-RADS is identifying one or more local champions within the radiology group or department. In a survey of 232 non-academic radiologists, the most common barriers to LI-RADS adoption were a lack of familiarity and referring clinicians not using the algorithm [17]. Local champions who have developed expertise and familiarity with LI-RADS can serve as a resource for other radiologists in the department as they develop their own familiarity. Through impromptu reading room consultations, phone calls or virtual platform screen sharing sessions, these experts can help with day-to-day questions or more challenging applications of the different LI-RADS algorithms. Champion radiologists should be facile with all LI-RADS Core documents and may also direct novice users to on-line resources, such as LI-RADS calculators that provide interactive tables and templates.

Local champions also act as liaisons with referring clinicians. While almost 90% of surveyed physicians (radiologists and referring clinicians) in North America preferred the use of LI-RADS to no or other standardized reporting systems, open communication between radiologists and referring clinicians is crucial to ensure that LI-RADS implementation aligns with local practice patterns [18]. Multidisciplinary treatment meetings provide an avenue of communication between champion radiologist and referring clinicians to address questions and concerns with LI-RADS adoption. Their expertise with LI-RADS and dialogue with referring clinicians enable local champions to develop and maintain standardized reporting templates relevant to local practice.

An issue that arises is that there may be no local champion available at the institute for the implementation of LI-RADS particularly on the global level where institutes may be unfamiliar with the LI-RADS algorithms. The ACR has created a LI-RADS education program on the ACR website that should serve as a starting point to help introduce physicians to the LI-RADS algorithms [19]. There are also hands on workshops that occur at subspecialty conferences such as the Society of Abdominal Radiology. Additionally, the members of the ACR LI-RADS committee can serve as potential mentors for physicians at institutes that are hoping to start a LI-RADs program.

LI-RADS has both an Outreach & Education group which focuses on encouraging radiology departments, predominantly in North America, to adopt LI-RADS. This includes educational materials, encouraging development of a champion radiologists in each radiology department. As for international adoption of LI-RADS, there is a LI-RADS International Working group which has members in many countries around the world. LI-RADS has also been translated into 14 different languages for the benefit of the international community [20]. LI-RADS has also been accepted into other local, regional and international organizations such as Cancer Care Ontario which mandates the use of LI-RADS in radiology reports as part of its HCC pathway [21].

#### Structured reporting and templates

Structured reports have been shown to be more complete and clearer when developed with collaboration between referring providers and radiologists [22, 23, 22, 24]. Moreover, structured reports can facilitate the use of practice guidelines by incorporating standardized terminology [25]. For these reasons, structured reporting and templates are powerful tools in promoting adoption and adherence to reporting systems such as LI-RADS. Standardized templates can ensure that the key features are described and are clearly linked to the appropriate diagnostic category and management recommendation. In LI-RADS, this has been shown to improve the quality and consistency of HCC reporting compared to free text reports [5, 26]. In the authors’ experiences, structured reporting is instrumental in the successful adoption and sustained use of LI-RADS among radiologists [4]. The benefit is particularly evident for radiologists who are involved in multidisciplinary liver conferences. LI-RADS structured reports help radiologists efficiently prepare and ensure adherence to the HCC reporting standards that are expected by the multidisciplinary team. The use of structured reports also facilitates the dissemination of updates to the reporting system and is particularly helpful when reviewing subsequent follow-up exams [27]. Finally, structured reporting may facilitate the automated extraction of data in radiology reports for research or for quality improvement initiatives.

The LI-RADS CT/MRI manual, ACR website, ACR Assist, the SAR HCC-DFP website and this manuscript (*add supplemental resource with template*) offer sample structured templates for LI-RADS reporting that serve as a starting point for any group instituting standardized templates for LI-RADS [28]. Radiology departments can build structured templates and macros in the reporting system based on the LI-RADS template provided in the manual. For instance, nesting the “LI-RADS observation” macro as a picklist option in the liver section of standard abdominal template makes it easier for the radiologists to access and use the macro. Radiology reporting platforms are now building structured reporting templates and guidance directly into their systems. These can be leveraged to provide more actionable reporting.

**Fig. 2:**
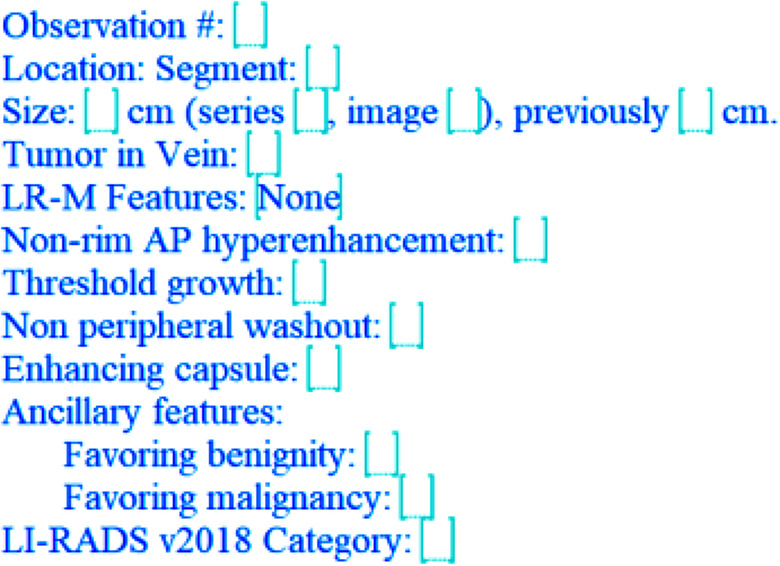
Lirads observation template

**Fig. 3:**
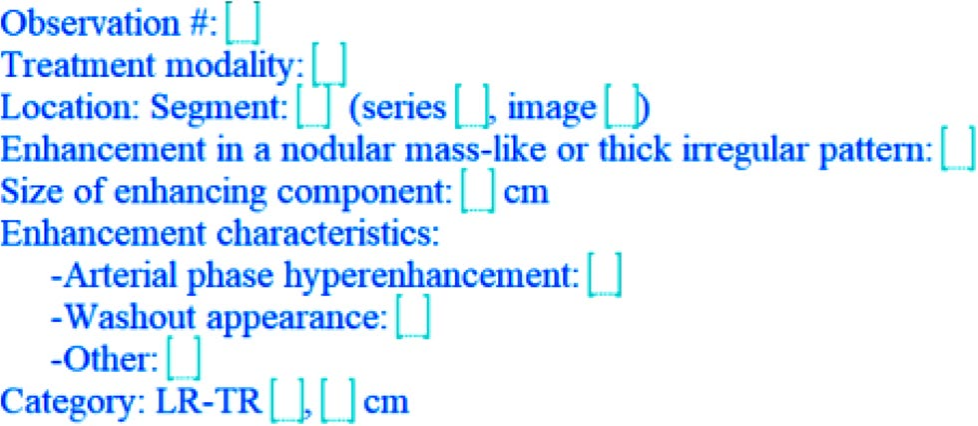
Lirads post-treatment template

For those that are more comfortable with free text reporting, LI-RADS also has a free text option that allows for the relevant information to be included [29] (See Figs. 2 and 3).

#### Challenges of LI-RADS adoption

LI-RADS faces several challenges that affect its broader adoption, particularly in non-academic and global settings. The number of different algorithms and the number of overall LI-RADS categories can be viewed as inefficient and daunting for some radiologists, leading to variability in adoption [8, 17]. Furthermore, a large list of ancillary features adds complexity and inefficiency to workflow and may account for 41% of nonacademic radiologists either sometimes, rarely, or never used the ancillary features to characterize a lesion [17]. The system’s specificity, while a strength, necessitates a corresponding loss of sensitivity that may not be suitable for all practice environments. To address these issues, simplifications of the algorithm have been proposed, aiming to maintain accuracy while easing use [8]. Moreover, the need for regular updates, keeping LI-RADS contemporaneous with developing literature, requires a commitment by users to continuously update their practice patterns and educate all stakeholders of substantive changes [8].

Some resistance to structured reporting stems from a perception of longer time to completion compared to free dictation [30]. While this may be true during the initial transition to structured reporting, the extra time can be justified by the improved quality and comprehensiveness of the report. Furthermore, it is typically a temporary issue that resolves once radiologists grow accustomed to the new template [31]. An additional cause of resistance is that the radiologists can feel constrained by the structured reports particularly for cases that follow atypical patterns that require more descriptive information [30]. In that regard, the LI-RADS manual chapter on reporting offers helpful tips and examples for reporting such scenarios, such as infiltrative appearance of HCC.

*Another challenge for HCC diagnosis that may lead to issues with adoption of LI-RADS is that the background fat or iron deposition in liver can make assessment more challenging for HCC with CT. However MRI is a good alternative in these patients*,* since subtraction imaging can help eliminate the background liver signal intensity and allow for a more clear assessment of the degree of enhancement* [9].

#### Overcoming internal barriers

It is important to recognize all team members as stakeholders for the successful implementation of a program. This can include radiology colleagues, imaging technologists, sonographers and trainees. As previously discussed, having a local radiologist champion is also paramount for the successful implantation.

Creating specific CT, MRI and US scan protocols on the machines with accompanied laminated cards may help ensure that the appropriate phases of imaging and anatomy are included for at risk / target populations. Working closely with lead technologists and providing annual radiologist lead educational opportunities can help overcome the barrier of incomplete or inappropriately performed exams.

For trainees, options can include workstation teaching, cue-cards, placards, online resources, and local trainee lectures or teaching rounds. Attending multidisciplinary hepatobiliary conference or HCC clinic enables trainees to realize the importance of using the precise terminology outlined in LI-RADS across modalities. Furthermore, McCann et al. showed that faculty are more likely to use LI-RADS standardized reporting templates when residents initially select the LI-RADS template [4].

#### Overcoming external barriers

The main external barrier to adopting LI-RADS is the lack of use by referring clinicians or endorsement by regional practice guidelines described in a recent international survey of HCC imaging [32]. In 2018, LI-RADS CT/MRI algorithms were integrated into AASLD guidance for HCC diagnosis, staging and management [8]. However, AASLD guidelines need to be championed by radiologists to bridge the gap of knowledge of HCC between primary care and hepatology.

A vital part of implementation of LI-RADS is providing screening and surveillance of patients at high risk for primary liver tumors. Ultrasound surveillance every 6 months is supported by AASLD as the mainstay of liver surveillance due to widespread access and lower costs compared to CT or MRI [33]. Modeling after successful efforts like Lung Cancer screening protocols may aid in establishing and incorporating LI-RADS Ultrasound Surveillance into practices. Providing definitions to LI-RADS categories and follow-up recommendations are helpful to referring clinicians not familiar with LI-RADS.

Currently, most primary care physicians in large health care networks utilize electronic medical records to trigger screening of hepatitis B on best practice advisory [34]. LI-RADS Ultrasound Surveillance can be incorporated similarly to lung screening into a health maintenance module based on demographics, and Hepatitis B status as well as the ICD 10 of cirrhosis to set a “target” population [33]. This could improve upstream screening based on accessibility and familiarity of ultrasound as a modality for primary care.

Setting up a chronic liver disease service line with multidisciplinary stakeholders requires service champions in Primary Care, Radiology and Hepatology. Downstream stakeholders can include Interventional Radiology, Medical Oncology, Radiation Oncology and Hepatobiliary/Transplant Surgery.

### Educational initiatives

Initial and continuing education are key to the successful implementation and optimal ongoing use of LI-RADS. As with any initiative, notifying all stakeholders a priori is crucial for the success of the program. As previously stated, this includes referring providers, pathologists, radiologists, technologists, multidisciplinary care coordinators, trainees and patients. Providing educational lectures, reference material and opportunities for feedback are all keys to success.

#### Education of referring providers

Providing didactic lectures and educational opportunities at grand rounds or other meetings of non-radiology stakeholders is important. Participating in discussion panels, conferences and other venues related to other key specialties treating patients with or at risk for HCC allows familiarization of other health care providers with LI-RADS including the associated management recommendations.

#### Education of radiologists and trainees

This can include hands-on courses, didactic lectures as well as having radiologist “champions” in the department who act as mentors or helpers on challenging cases for radiologists who are less experienced or just starting to use LI-RADS. Radiology trainees should be integrated in this training workflow and have dedicated lectures as part of the curriculum to educate them about the appropriate use of LI-RADS. The ABR in its list of core concepts under the domain blueprint for Gastrointestinal Imaging lists “ Focal diseases of the liver, benign and malignant” which LI-RADS would fall under [35]. Discussion of anonymized cases at peer learning sessions as well as providing individual feedback based on multidisciplinary case conference review of patients discussed at those venues can be of value. Development of QI projects to assess adherence to LI-RADS in a radiology department as well as QI for accuracy of reports correlated with surgical findings could be considered as well.

#### Education of technologists

Without high quality images, especially obtaining the late hepatic arterial phase on CT and MRI, interpretation of liver observations will be negatively affected Thus, including technologists, and technologist champions in the implementation of LI-RADS and providing feedback is key. Technologist- specific peer review or peer learning cases related to image quality can be helpful to show examples of optimal and suboptimal imaging and ways to maintain or improve image quality in the future.

#### Education of patients

Patients are able to view their reports, often before they meet with the health provider who requested imaging. Thus, having resources available for patients to better understand their imaging report related to the liver and potential HCC is important. There are websites available which can explain the significance of the findings and explain them in a non-medical way, such that the majority of individuals can understand. Artificial intelligence also has the potential to provide layperson explanations of imaging reports to patients so that they can participate more effectively in decision-making regarding their health.

Other educational initiatives for upstream stakeholders should address:

(1) patients at high risk require surveillance every 6 months if US-1 or negative screening exam.

(2) Actionable reporting triggering either a 3-month ultrasound subthreshold observation (US-2) or a multiphase exam for screen detected observation that exceeds 1 cm (US-3).

Championing a chronic liver disease service line, including adoption of US surveillance enables early diagnosis and helps to create a pathway for primary care providers to easily access hepatology, interventional radiology and downstream multidisciplinary care through the CT/MR diagnostic LI-RADS algorithm.

## Maintaining the use of LI-RADS

Once LI-RADS has been implemented in a radiology department, regular evaluation of its use is key to continue to maintain its value within the medical community. This includes peer learning opportunities for technologists and radiologists, while continuing to engage stakeholders caring directly for patients at risk for HCC, to provide constructive feedback.

Multidisciplinary case conferences (MDCs) are excellent sources of feedback for radiologists. This feedback can be provided either directly by the radiologist attending MDC or through peer learning activities [27]. The American College of Radiology Peer Learning website provides helpful tips for such endeavors (https://www.acr.org/Practice-Management-Quality-Informatics/Peer-Learning-Resources).

Quality improvement projects should also be undertaken. Plan-do-study-act (PDSA) cycles can be implemented for many QI projects [36]. Examples include technologist-led image quality assessments, with emphasis on proper timing to acquire the late arterial phase for both CT and MRI.

Departmental QI projects can evaluate adherence to standard template report use for various LI-RADS algorithms by radiologists and learners and can monitor for changes in adherence. Additional department-wide QI projects may include:

a) Assessment of inter-observer variability of LI-RADS categories.

b) Correlation between final management pathway at MDC and original LI-RADS category assigned.

c) Evaluating accuracy of LI-RADS categories in subgroups of patients who undergo biopsy, surgery or transplant.

Feedback loops in the form of QI create opportunities for technologists to acquire high quality images and for radiologists to hone their skills applying LI-RADS over time.

## Future directions

**Fig. 4:**
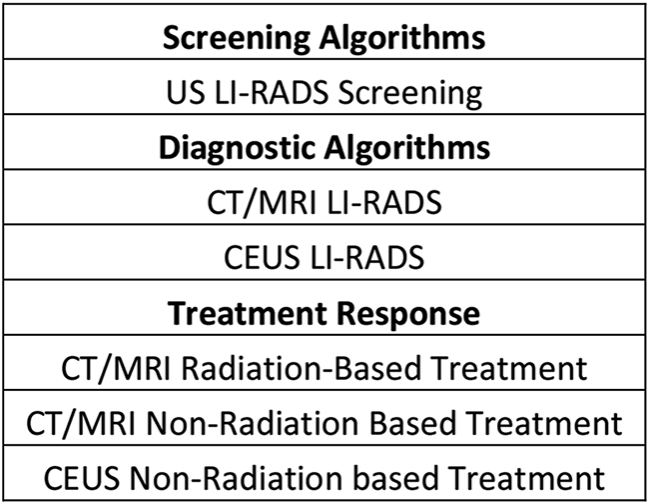
Current LI-RADS algorithms

**Fig. 5:**
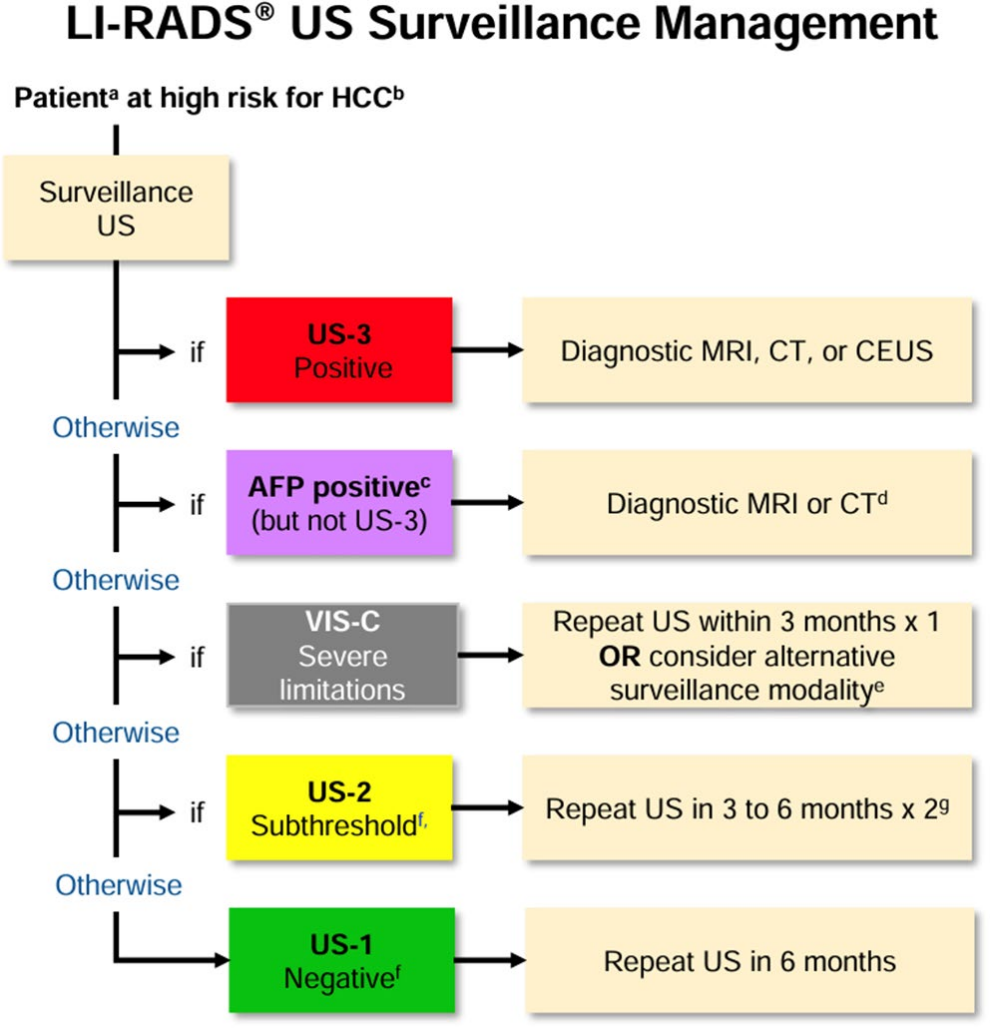
LI-RADS US surveilance 2024 core algorithm [38]

**Fig. 6:**
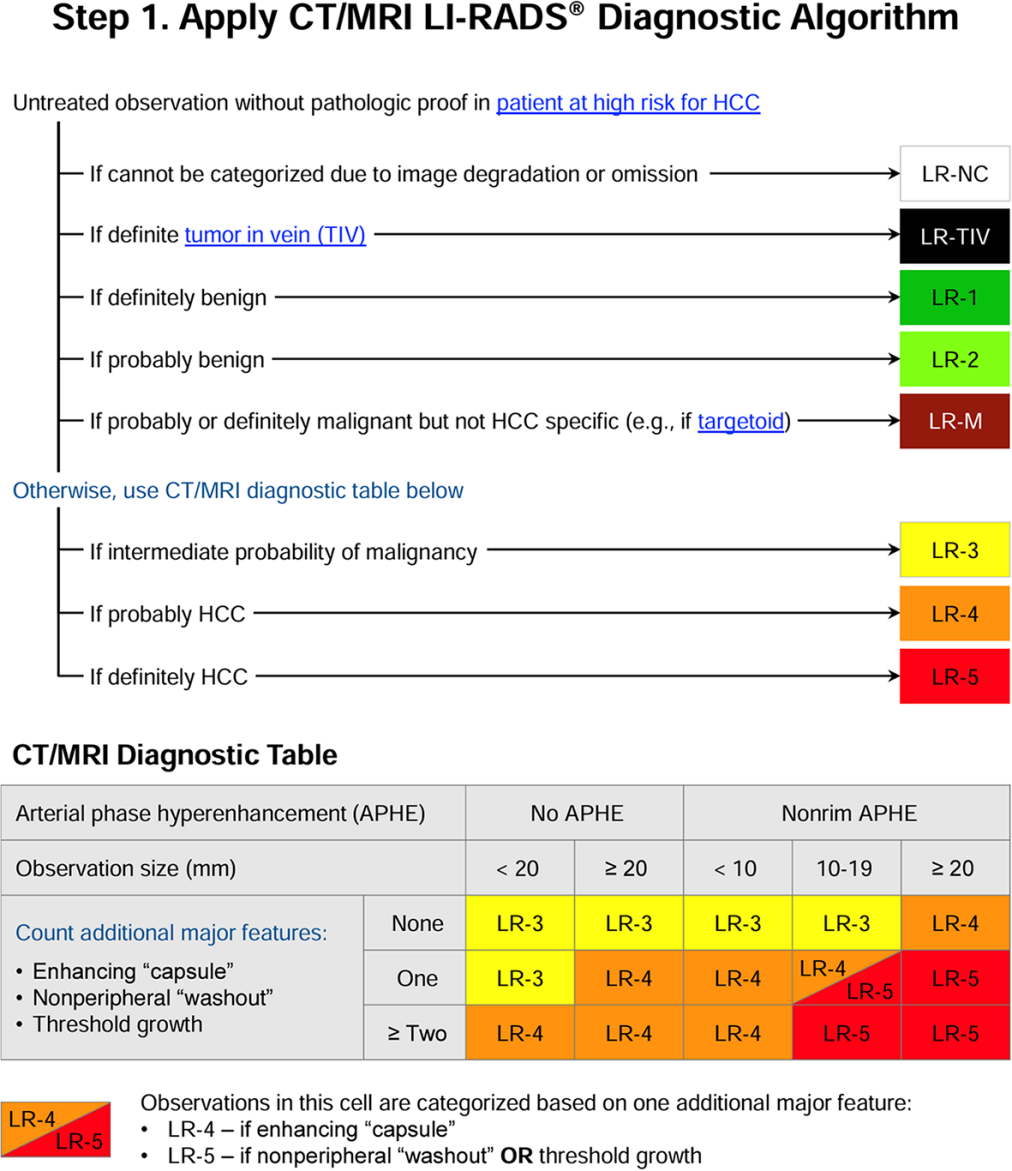
CT/MRI LI-RADS diagnostic algorithm v2018 [38]

**Fig. 7:**
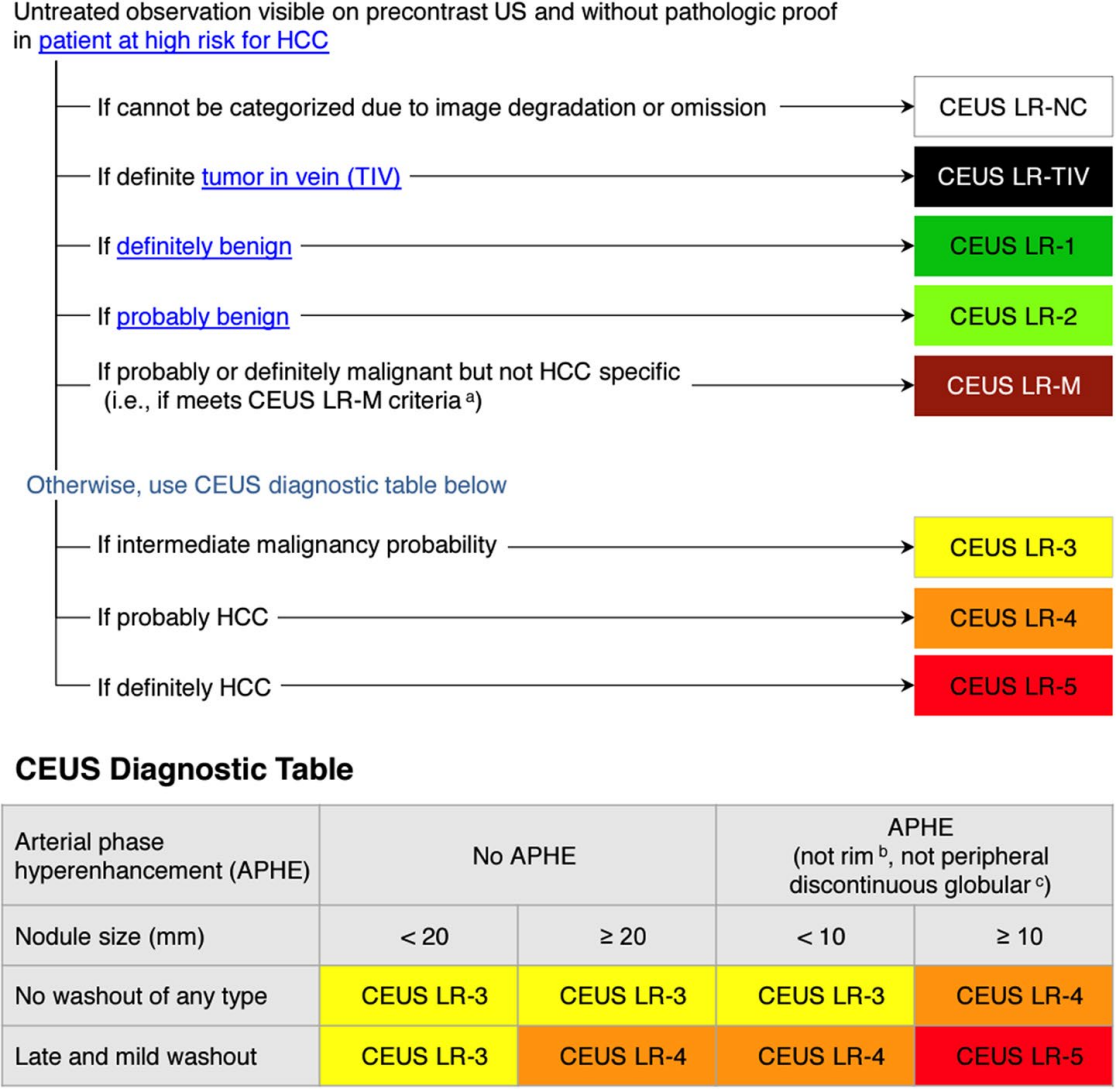
CEUS LI-RADS v2017 core [38]

**Fig. 8:**
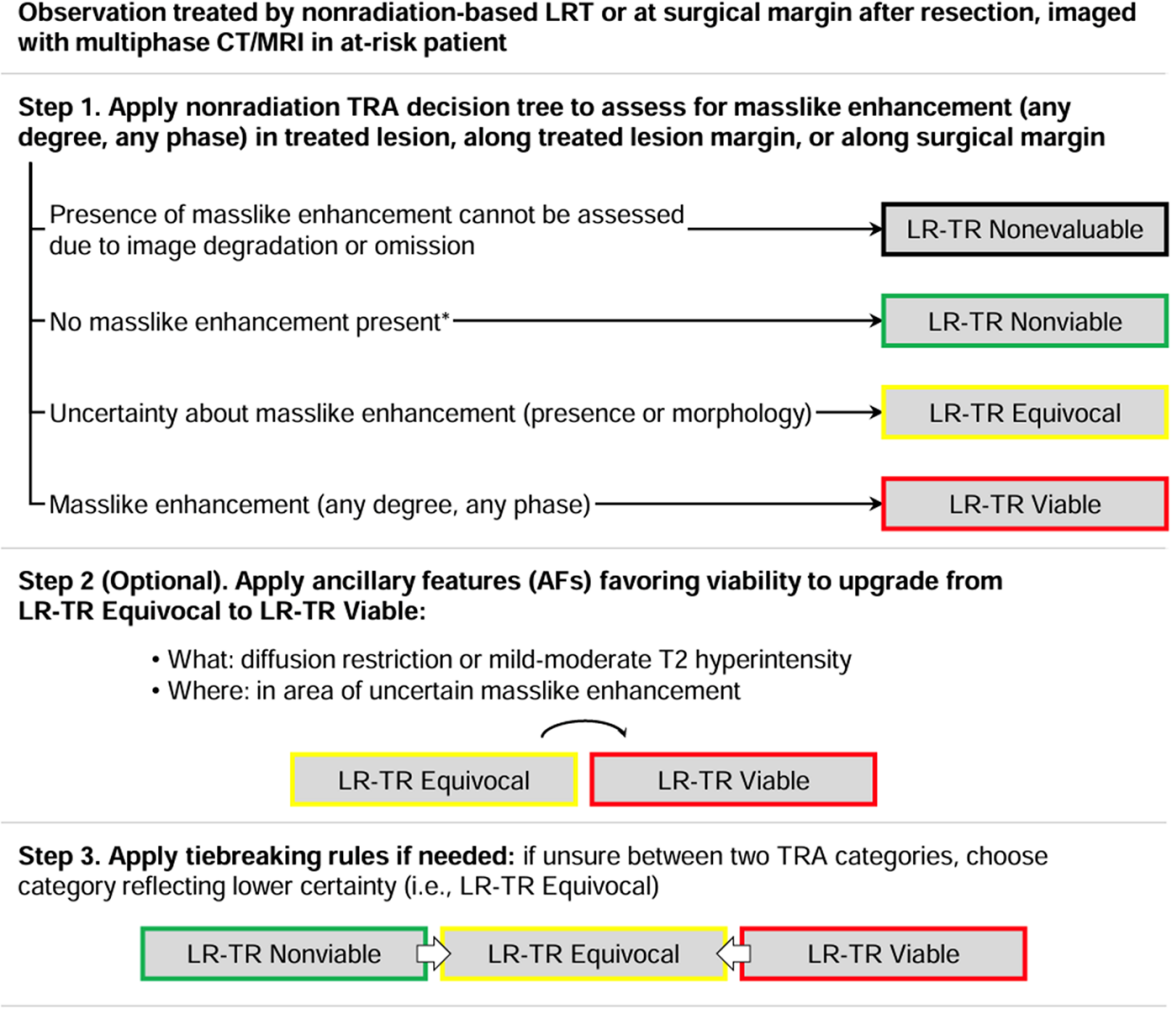
LI-RADS CT/MRI non-radiation TRA v2024 core [38]

**Fig. 9:**
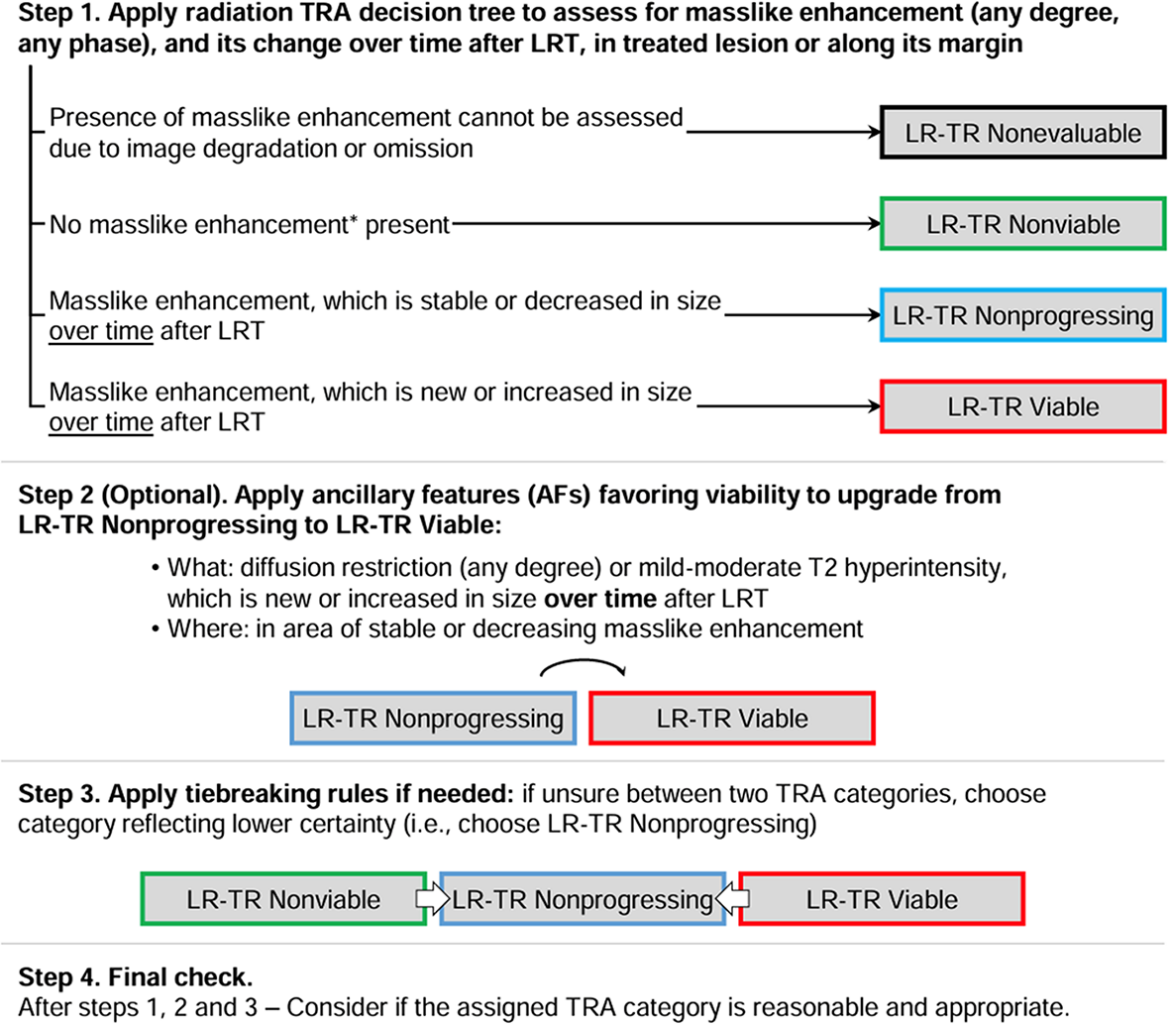
LI-RADS CT/MRI radiation TRA v2024 core [38]

**Fig. 10:**
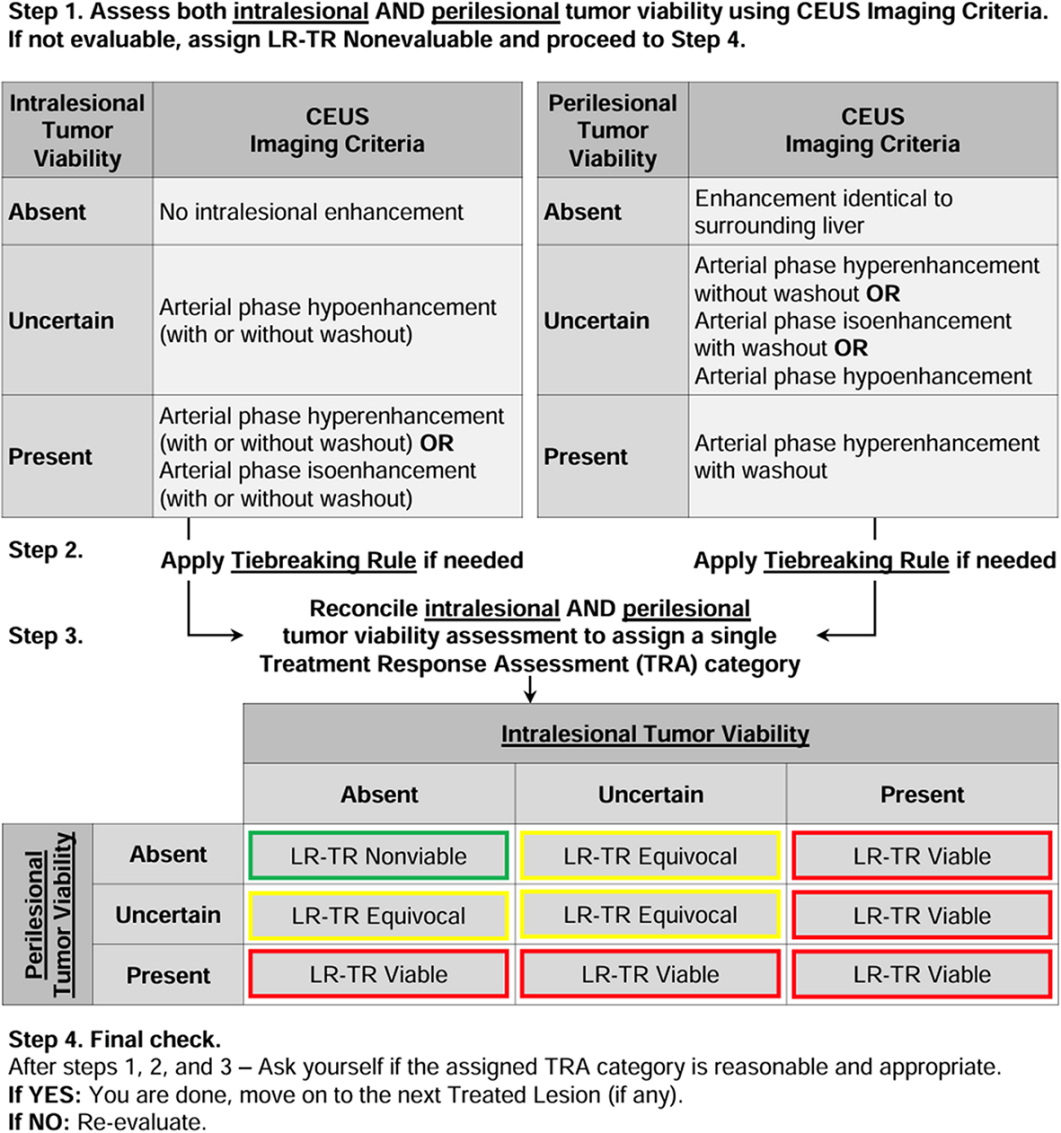
LI-RADS CEUS non-radiation TRA v2024 [38]

Since 2011, LI-RADS has undergone several updates and additions as outlined in Fig. 4 [37]. Each update and additional algorithm have introduced new information, responded to user feedback, and implemented improvements. Balancing the integration of new insights and technological progress with the need for stability is crucial. LI-RADS has established short-term and long-term goals to ensure its ongoing growth and effectiveness, with a plan to revise the diagnostic algorithms every 3 to 5 years as new knowledge emerges [8, 37] (See Figs. 5, 6, 7, 8, 9 and 10).

The future directions of LI-RADS are influenced by evolving clinical needs, advances in technology, and continuous research in liver imaging. As our understanding of HCC and its risk factors continues to evolve, LI-RADS is expected to incorporate more personalized risk stratification approaches. This may include integrating imaging features associated with tumor grade, treatment response and patient outcome. Personalized risk assessments could lead to more tailored surveillance protocols and intervention strategies, ultimately improving patient outcomes. Key future developments are likely to focus on linking LI-RADS categories with patient outcomes, thereby refining the system’s effectiveness based on real-world results. Engaging in further studies and clinical trials is crucial to validate and improve the accuracy of LI-RADS categories [37]. Utilizing data analytics to analyze extensive imaging data could uncover new insights that refine LI-RADS categories and applications. Establishing and maintaining comprehensive databases of liver imaging findings will facilitate ongoing updates and improvements based on a diverse set of patient data [8, 37].

One of the key areas for future development in LI-RADS is integration of prognostic imaging features and prediction of response. Current LI-RADS algorithms provide an accurate noninvasive diagnosis of HCC through a combination of imaging features in high-risk patients, but do not include any features that could predict outcomes and treatment responses. Several key imaging features have been associated with outcomes after treatment, for examples tumor margin, peritumoral hyperenhancement, peritumoral HBP hypointensity, non-targetoid feature and arterial phase engorged vessels [39, 40]. Integration of key prognostic imaging features will enable LI-RADS to provide valuable information to guide treatment selection, in addition to diagnosis [41].

Another area of potential improvement resides in the LI-RADS Treatment Response (TR) algorithm. It is a relatively young system, initially released in 2017, and has moderate inter-reader agreement [2]. The LI-RADS TR algorithm was not applicable for post radiation-based therapies until the recent release of post radiation treatment response algorithm LI-RADS CT/MRI Radiation TRA v2024, which includes 3 categories of LR-TR nonviable, LR-TR nonprogressing and LR-TR viable. Large cohort validation data are needed to support the accuracy of this relatively new algorithm. Global educational outreach on this algorithm is needed to help with more appropriate application of the LI-RADS TR system and to improve inter-reader agreement.

In addition to previously discussed goals, LI-RADS aims to leverage emerging technologies to more accurately define its target population and enhance both the precision and usability of the system. The integration of artificial intelligence (AI) in radiology is expanding rapidly. Potential AI applications within LI-RADS include lesion detection, tracking lesions over multiple exams, characterizing imaging features, and automating LI-RADS classifications. Natural language processing (NLP) could be used to extract data from reports and generate structured reports automatically. The creation of large imaging and clinical data repositories will support AI training, enhance algorithm refinement, and ultimately enable detailed assessments that integrate clinical, laboratory, and imaging data [8, 37].

### Summary

The adoption of LI-RADS in standardized reporting of liver lesions in patients at risk for hepatocellular carcinoma has enhanced communication between radiologists and care teams including radiologists, hepatologists, oncologists and surgeons [8]. Although some barriers to adoption do remain, the continuous evolution of LI-RADS through feedback from users has helped to improve the practicality of its adoption. Further work is needed through educational initiatives, supporting radiology champions, and technological advancements to help promote the continued advancement of LI-RADS with the goal of providing the best care to patients.

## Data Availability

No datasets were generated or analysed during the current study.
